# Sequence Types of *Cryptococcus neoformans* and Their Associations with Clinical Characteristics and Outcomes of AIDS Patients with Cryptococcal Meningitis in Southern China

**DOI:** 10.3390/pathogens15060605

**Published:** 2026-06-05

**Authors:** Chenfeng Li, Yurong Zhang, Yingchun Ke, Yeyang Zhang, Meijun Chen, Xingru Tao, Pengle Guo, Jingliang Chen, Xiaoping Tang, Weiyin Lin, Linghua Li

**Affiliations:** 1 Infectious Disease Center, Guangzhou Eighth People’s Hospital, Guangzhou Medical University, Guangzhou 510440, China; 2 Guangzhou Medical Research Institute of Infectious Diseases, Guangzhou 510440, China; 3 The Third Affiliated Hospital of Sun Yat-sen University (Yuedong Hospital), Meizhou 514700, China; 4 Department of Clinical Laboratory, Guangzhou Eighth People’s Hospital, Guangzhou Medical University, Guangzhou 510440, China; 5 Institute of Infectious Disease, Guangzhou Eighth People’s Hospital, Guangzhou Medical University, Guangzhou 510440, China

**Keywords:** HIV/AIDS, *Cryptococcus neoformans*, cryptococcal meningitis, clinical characteristics, MLST, antifungal susceptibility, prognosis

## Abstract

This study investigated the molecular epidemiological characteristics of *Cryptococcus neoformans* (*C. neoformans*) isolates from AIDS patients with cryptococcal meningitis (CM) in southern China and examined their associations with clinical features and outcomes. A total of 100 clinical isolates were identified by MALDI-TOF MS and genotyped by multilocus sequence typing (MLST). Antifungal susceptibility to five agents was assessed using the FUNGUS 3 system. Baseline demographic, clinical manifestations, radiological, and laboratory data were collected from the corresponding 100 patients, and outcomes were evaluated at weeks 4, 12, 24, and 48. Seven sequence types (STs) were identified: ST5 (83/100, 83.0%), ST4 (5/100, 5.0%), ST31 (3/100, 3.0%), ST43 (1/100, 1.0%), ST93 (4/100, 4.0%), ST395 (1/100, 1.0%), and a presumptive novel ST685 (3/100, 3.0%). Most patients were male (80.0%), and headache was the most common symptom (85.0%). Susceptibility rates for 5-flucytosine, amphotericin B, fluconazole, itraconazole, and voriconazole were 98.9% (94/95), 71.9% (69/96), 82.3% (79/96), 59.4% (41/69), and 86.8% (59/68), respectively. Cumulative mortality reached 16%, 33%, 37%, and 39% at weeks 4, 12, 24, and 48. No significant differences were observed between 83 patients infected with ST5 and 17 patients with non-ST5 in clinical presentations or antifungal susceptibility. However, patients infected with ST5 exhibited consistently better survival rates across all time points; the 12-week non-survival rates were highest for patients infected with ST93 and ST395. Accordingly, ST5 is the dominant sequence type of *C. neoformans* in HIV-associated CM in southern China; meanwhile, non-ST5 types could have worse prognosis, indicating ST sequence typing may act as a prognostic biomarker in AIDS patients with CM.

## 1. Introduction

*Cryptococcus* species are opportunistic pathogenic fungi widely distributed in the environment worldwide. The genus primarily comprises two major species complexes of clinical importance: *Cryptococcus neoformans* (*C. neoformans*) and *Cryptococcus gattii* (*C. gattii*). Among them, *C. neoformans* has been designated by the World Health Organization (WHO) as a “critical-priority” fungal pathogen due to its substantial global disease burden [1,2]. Cryptococcosis caused by *C. neoformans* manifests with diverse clinical presentations in humans, most commonly involving the lungs and the central nervous system (CNS) [2]. Characterized by marked neurotropism, *C. neoformans* readily invades the central nervous system and causes life-threatening cryptococcal meningitis (CM). Globally, CM is estimated to cause approximately 112,000 deaths annually [3]. Despite the widespread implementation of antiretroviral therapy (ART), HIV-associated CM remains a major contributor to AIDS-related mortality [4].

The geographic distribution, host susceptibility, and sequence type (ST) profiles of *Cryptococcus* exhibit marked regional variability. To facilitate global epidemiological comparisons, the multilocus sequence typing (MLST) scheme recommended by the International Society for Human and Animal Mycology (ISHAM) working group—based on seven housekeeping loci (CAP59, GPD1, LAC1, PLB1, SOD1, URA5, and IGS1)—provides a standardized framework for defining nine major molecular types, comprising VNI, VNII, VNB, VNIII, and VNIV within *C. neoformans*, and VGI, VGII, VGIII, and VGIV within *C. gattii* lineages [5]. In China, cryptococcal infections among HIV-positive individuals appear to exhibit a relatively homogeneous genotype distribution. Studies from Zhejiang and Taiwan have consistently reported a predominance of the VNI molecular type, particularly sequence type ST5 [6,7,8,9]. Compared with the global MLST dataset, the Asian *C. neoformans* population demonstrates relatively limited genetic diversity [10]. Globally, distinct regional patterns have been observed: ST93 predominates among HIV-associated cases in sub-Saharan Africa, particularly in the Democratic Republic of the Congo; ST23 and ST63 are more common in Europe; whereas ST31 and ST5 are frequently reported in India and Southeast Asia [10,11,12,13,14]. In contrast, genotyping data for HIV-infected patients in China remain limited, although available evidence suggests that ST5 is the dominant sequence type [8].

In southern China, where cryptococcosis remains highly prevalent, systematic investigations integrating MLST-based sequence typing and antifungal susceptibility data are still lacking. Although molecular epidemiological studies of *Cryptococcus* have expanded in recent years, most have focused primarily on sequence type characterization and routine antifungal susceptibility testing, with limited evaluation of baseline clinical characteristics or long-term outcomes across different sequence types, particularly in AIDS patients with CM [15,16].

To address these gaps, we conducted a retrospective cohort study in southern China to characterize the MLST-defined sequence types of *C. neoformans* isolates and assess their associations with baseline clinical features and long-term outcomes. This study aims to enrich the knowledge of the molecular epidemiology of *C. neoformans* in southern China and discover a new prognostic biomarker for AIDS patients with CM.

## 2. Materials and Methods

### 2.1. Study Design and Patient Population

A total of 100 well-preserved clinical *Cryptococcus* isolates were separately obtained from cerebrospinal fluid (CSF) cultures of 100 hospitalized AIDS patients with CM. All patients were admitted to the Infectious Disease Center of Guangzhou Eighth People’s Hospital, Guangzhou Medical University, between 23 March 2020 and 28 October 2024. The inclusion criteria were age ≥ 18 years, confirmed human immunodeficiency virus (HIV) infection [17], diagnosis of HIV-associated CM, and a positive CSF culture for *Cryptococcus* with preserved isolates available for subsequent analysis. CM was diagnosed according to previously published criteria [18], based on clinical manifestations consistent with meningitis, and positive culture of *Cryptococcus* in CSF. The exclusion criteria were pregnancy, lactation, and concomitant other CNS infections, including bacterial, viral, tuberculous, toxoplasma gondii, or non-cryptococcus fungal meningitis.

### 2.2. Clinical Data Collection

Clinical and demographic data were retrospectively obtained from electronic medical records and supplemented by telephone follow-up. Variables included age, gender, presenting symptoms, time from symptom onset to diagnosis, meningeal signs, CD4^+^ T-cell counts, CD4^+^/CD8^+^ ratios, CSF opening pressure, neuroimaging findings, antifungal therapy, and clinical outcomes. Patients were followed at baseline and at weeks 4, 12, 24, and 48. Data were entered into a standardized database using double data entry and independently verified by two investigators to ensure accuracy.

### 2.3. Fungal Isolation and Identification

CSF specimens were processed by centrifugation, and the supernatant was discarded. The sediments were initially screened by India ink staining. Encapsulated round or oval yeast-like cells were considered suggestive of *Cryptococcus* spp. CSF specimens were then inoculated onto Sabouraud dextrose agar and incubated at 28 °C for 24–72 h to obtain pure cultures. Species-level identification was further performed using the VITEK MS MALDI-TOF MS system (bioMérieux, Craponne, France) in accordance with the manufacturer’s instructions. Briefly, a small amount of a freshly grown single colony was transferred onto the designated spot of a VITEK MS target slide using a 1-μL plastic inoculation loop, with care taken to avoid agar carryover. The sample was overlaid with 0.5 μL of 25% formic acid and air-dried at room temperature. Thereafter, 1 μL of α-cyano-4-hydroxycinnamic acid (CHCA) matrix solution was applied and air-dried. After barcode scanning and acquisition of the target slide information, the slide was loaded into the VITEK MS instrument for spectral acquisition and database-based species identification. The confirmed isolates were stored at −80 °C for long-term preservation.

### 2.4. Multilocus Sequence Typing

The isolates were first revived from storage, and genomic DNA was subsequently extracted using the MasterPure™ Yeast DNA Purification Kit (Lucigen, Middleton, WI, USA). MLST was performed according to the ISHAM consensus scheme. Locus-specific primers were designed for seven housekeeping genes (*CAP59*, *GPD1*, *IGS1*, *LAC1*, *PLB1*, *SOD1*, and *URA5*), followed by PCR amplification (Table 1). The amplified products were verified by agarose gel electrophoresis and subsequently sequenced. STs were assigned by comparison with the Fungal MLST database.

### 2.5. Antifungal Susceptibility Testing

Antifungal susceptibility testing was performed using the ATB FUNGUS 3 system (bioMérieux, France) according to the manufacturer’s instructions. Five antifungal agents were tested: 5-flucytosine (5-FC), amphotericin B (AMB), fluconazole (FLC), itraconazole (ITR), and voriconazole (VRC). The minimum inhibitory concentrations (MICs) were determined and categorized as susceptible (S), intermediate (I), or resistant (R) according to CLSI guidelines; for subsequent analysis, intermediate and resistant isolates were combined into a non-susceptible group.

### 2.6. Statistical Analysis

Statistical analysis was conducted using SPSS version 26.0 (IBM Corp., Armonk, NY, USA) and GraphPad Prism version 8.2.1 (GraphPad Software, San Diego, CA, USA). Continuous variables were expressed as medians with interquartile ranges and compared using the Mann–Whitney U test or independent *t*-test, as appropriate. Categorical variables were analyzed using the chi-square test or Fisher’s exact test. Survival analysis was performed using the Kaplan–Meier method, and survival differences between groups were assessed using the log-rank test. *p* < 0.05 was considered statistically significant.

## 3. Results

### 3.1. MLST of 100 Cryptococcus Isolates

**Figure 1:**
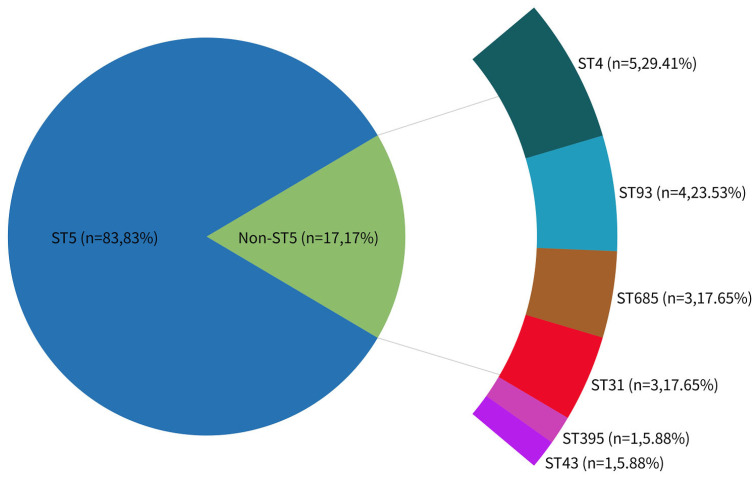
Distribution of MLST sequence types among 100 *Cryptococcus neoformans* Isolates. ST5 was the predominant sequence type (83%, 83/100), while non-ST5 types accounted for 17% (17/100). The distribution of non-ST5 types is shown in the inset.

The 100 *Cryptococcus* isolates were recovered from the CSF of AIDS patients with CM, and all isolates were identified as *C. neoformans* by MALDI-TOF. Based on MLST analysis, 99 of 100 *C. neoformans* isolates were VNI and 1 was VNII. A total of seven STs (Table 2) were identified, of which ST5 was the predominant type (83/100, 83.0%), followed by ST4 (5/100, 5.0%), ST93 (4/100, 4.0%), ST31 (3/100, 3.0%), ST43 (1/100, 1.0%), ST395 (1/100, 1.0%), and a presumptively novel ST685 (3/100, 3.0%) (Figure 1).

### 3.2. Comparison of Clinical Characteristics of AIDS Patients with CM Infected by ST5 or Non-ST5 C. neoformans Strains

The baseline demographic and clinical characteristics of 100 AIDS patients with CM are summarized in Table 3. The overall cohort was predominantly male (80.0%) with a median age of 40 years (IQR, 30–47). Headache (85.0%), fever (58.0%), and nausea/vomiting (56.0%) were the most common presenting symptoms, while meningeal irritation (60.0%), disturbance of consciousness (23.0%), and convulsions (9.0%) represented the major neurological manifestations. Antifungal susceptibility testing was performed for 5-FC (n = 95), AMB (n = 96), FLC (n = 96), ITR (n = 69), and VRC (n = 68). Overall, the isolates showed high susceptibility to 5-FC (94/95, 98.9%) and VRC (59/68, 86.8%), followed by FLC (79/96, 82.3%) and AMB (69/96, 71.9%), whereas susceptibility to ITR was relatively lower (41/69, 59.4%).

The baseline characteristics and antifungal susceptibility profiles were generally comparable between the ST5 and non-ST5 groups. No statistically significant differences were found in demographic features, clinical manifestations, time from symptom onset to diagnosis, laboratory findings, neuroimaging results, or susceptibility to the five antifungal agents between the two groups (all *p* > 0.05). A higher frequency of convulsion and a trend toward elevated CSF intracranial pressure were observed in the non-ST5 group, but neither difference reached statistical significance (*p* = 0.067 and *p* = 0.076, respectively).

### 3.3. Survival Analysis of AIDS Patients with CM Infected by ST5 or Non-ST5 C. neoformans Strains

**Figure 2:**
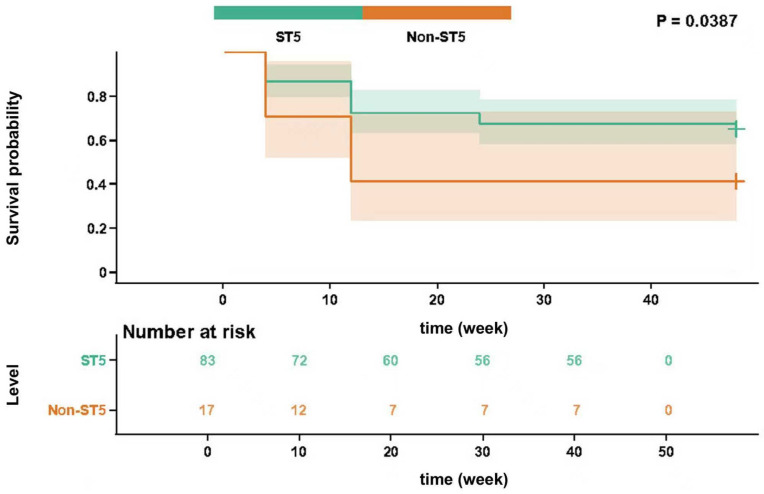
Kaplan-Meier survival curves of AIDS patients with CM infected with *Cryptococcus neoformans* ST5 and non-ST5 types over a 48-week follow-up. Survival was significantly higher in the ST5 group than in the non-ST5 group (*p* = 0.0387). Shaded areas represent 95% confidence intervals. The numbers at risk at each time point are shown below the plot.

The cumulative mortality reached 16%, 33%, 37%, and 39% at weeks 4, 12, 24, and 48 for 100 patients. Significantly higher overall survival in patients with ST5 was observed by Kaplan–Meier survival analysis, compared with those with non-ST5 types (*p* = 0.0387; Figure 2). The distribution of ST types within the survival data was significantly different at 12 weeks (*p* = 0.038; Table S1). The proportions of patients infected with ST93 and ST395 were higher in the non-survival group than in the survival group (Table S1).

### 3.4. Discovery of the Novel ST685 Sequence Type Associated with Fluconazole Resistance and Variable Prognosis

Three isolates were identified as a novel sequence type, ST685, which has not been previously reported in the ISHAM MLST database. Notably, one isolate was FLC resistant. The patient harboring this isolate showed sustained clinical improvement across four scheduled follow-up visits and remained alive at 48 weeks. This suggests that the newly identified ST685 sequence type may be associated with reduced FLC susceptibility, although its clinical significance remains unclear and warrants further investigation.

## 4. Discussion

*C. neoformans*, the primary etiological agent of cryptococcosis, poses significant clinical challenges due to its complex pathogenic mechanisms. Host damage occurs at multiple levels, including molecular, cellular, and tissue levels, through processes such as non-lytic exocytosis, organelle dysfunction, phagosomal membrane damage, and cytoskeletal disruption. These mechanisms impair immune cell function and compromise endothelial barriers, facilitating fungal dissemination [19]. A diverse range of molecular typing techniques has been applied to characterize this pathogen, among which MLST has become the gold standard for ongoing strain typing and epidemiological surveillance of *Cryptococcus* [20,21].

In this retrospective cohort, *C. neoformans* ST5 was overwhelmingly predominant, accounting for 83% of isolates. This finding aligns with previous reports from China, whether in HIV-positive patients or in HIV-negative patients, where ST5 is the dominant lineage, as well as with observations from Malaysia, Brazil, and South Africa. However, this pattern contrasts with other regions: ST93 is predominant among HIV-associated cryptococcal infections in sub-Saharan Africa and Latin America, whereas ST23 and ST63 are more common in Europe, indicating that sequence type prevalence is geographically associated [22,23,24]. In our cohort, the remaining 17% of isolates comprised fewer common types (ST4, ST31, ST43, ST93, ST395) and one novel sequence type, designated ST685. Of note, our study revealed less sequence type diversity in southern China, consistent with a previous Indian study that demonstrated lower genetic diversity among clinical isolates in Asia compared to the global MLST database [25]. This supports the notion that regional *C. neoformans* populations may exhibit distinct genetic structures.

In this study, AIDS patients with CM presented with typical but nonspecific neurological symptoms, particularly headache, accompanied by a high prevalence of meningeal irritation, reflecting the characteristic manifestations of CM driven by elevated intracranial pressure. In terms of antifungal susceptibility, the resistance rates to FLC, ITR, and 5-FC were 4.2%, 1.4%, and 1.1%, respectively, while no resistance was observed to AMB or VRC. Among the antifungal agents tested, FLC exhibited the highest resistance rate. This pattern aligns with global trends showing a gradual rise in FLC resistance among *Cryptococcus* species. Consistent with a Chinese study (2013–2017) reporting increasing FLC MICs over time [9], and global surveillance data showing FLC resistance rising from 7.3% (1997–2000) to 10.9% (2001–2007) [14], our findings underscore the need for ongoing antifungal susceptibility surveillance. To the best of our knowledge, this is the first study from South China to provide a relatively comprehensive comparison of clinical manifestations between ST5 and non-ST5 infections in AIDS patients with CM, with a particular focus on CM-related neurological features. Most baseline clinical characteristics were comparable between the two groups. Although convulsions were more frequent and CSF intracranial pressure tended to be higher in the non-ST5 group, these differences did not reach statistical significance and should therefore be interpreted cautiously. Whether these non-significant trends are related to the poorer outcomes observed in the non-ST5 group requires validation in larger cohorts. Simultaneously, susceptibility analysis of five clinically relevant antifungal agents showed no significant differences between the two groups. However, our observation of no association between sequence type and antifungal susceptibility aligns with a study from Brazil, which reported that all isolates were susceptible to the antifungal agents tested and found no correlation between antifungal susceptibility and sequence type [26,27]. The lack of sequence type–phenotype association in our cohort may be partly attributable to the uniformly advanced immunosuppression in our study population, all of whom were HIV-positive. In the setting of advanced HIV infection, profound host factors—such as severe CD4-positive T-cell depletion and elevated fungal burden—are likely to drive the clinical presentation, potentially overshadowing any subtle differences in virulence among cryptococcal STs. Future prospective multicenter studies with larger cohorts and more comprehensive clinical and microbiological data are needed to further clarify the relationship between sequence types and fungal phenotypic characteristics.

AIDS patients with CM have a high mortality rate, and even in clinical trials the 10-week mortality remains approximately 40% [4]. Both groups received antifungal therapy and routine medical dehydration therapy, and no surgical intervention was performed to reduce intracranial pressure. While baseline features were similar, patient outcomes differed significantly by *Cryptococcus* sequence type. In this study, the cumulative mortality reached 16%, 33%, 37%, and 39% at weeks 4, 12, 24, and 48, respectively. Our results showed significantly higher survival in patients infected with the dominant ST5 strain compared with those infected with non-ST5 strains (*p* = 0.0387). In particular, patients infected with ST93 or ST395 showed poor short-term outcomes in our cohort. This finding contrasts with two previous studies from China, which suggested that HIV-associated cryptococcosis caused by ST5 lineages is more difficult to diagnose and manage than that caused by non-ST5 lineages [15,16]. However, in our cohort, no significant difference was observed in the time to diagnosis from symptom onset between the two groups (median [IQR]: 18 [10–30] vs. 16 [9–30] days; *p* = 0.340). The regional differences in prognosis between ST5 and non-ST5 strains in southern and northern China may be partly due to variations in the types of non-ST5 types circulating in different regions. In particular, patients infected with ST93 had the poorest short-term prognosis in our cohort. Among the four patients with ST93 infection, two died within 4 weeks and all had died by 12 weeks, which likely contributed substantially to the worse overall survival observed in the non-ST5 group. In contrast, a study from the Democratic Republic of Congo reported better outcomes associated with ST93, whereas non-ST93 sequence types were significantly associated with poorer outcomes (87.5% vs. 40%, *p* = 0.02) [13]. These observations further suggest that the relationship between Cryptococcus sequence types and clinical outcomes may differ geographically.

The identification of the novel sequence type ST685 represents a noteworthy finding in this study. To our knowledge, this sequence type has not previously been reported in the ISHAM MLST database, suggesting possible local evolution or underrecognized sequence diversity of *Cryptococcus* spp. in this region. Of particular interest, one ST685 isolate exhibited resistance to FLC, an important antifungal agent commonly used in the treatment of cryptococcosis. However, the corresponding patient showed sustained clinical improvement and survived through 48 weeks of follow-up. This observation suggests that in vitro antifungal susceptibility may not always directly predict clinical outcomes. The prognosis of cryptococcal meningitis is likely influenced by multiple factors, including host immune status, fungal burden, antifungal treatment regimen, treatment adherence, and management of intracranial pressure. Therefore, the clinical significance of ST685 and its possible association with reduced FLC susceptibility require further investigation in larger studies.

This study has several limitations. First, it was a single-center study with a relatively small sample size of 100 patients, which may limit the generalizability of the findings. In particular, the small number of patients infected with rare non-ST5 sequence types limited the statistical power to draw definitive conclusions regarding their clinical impact. Therefore, these findings should be interpreted cautiously. Nevertheless, because Guangzhou Eighth People’s Hospital is one of the major designated centers for HIV/AIDS care in Guangdong Province and receives patients from multiple regions of southern China, this cohort provides useful regional data. Larger multicenter studies are needed to validate these results. Second, the retrospective design may have resulted in incomplete clinical data and potential information bias, as clinical information was obtained from existing electronic medical records and telephone follow-up rather than through prospective standardized data collection. In addition, antifungal susceptibility testing was incomplete for some agents, which may limit the interpretation of susceptibility patterns. Prospective studies with standardized data collection and complete susceptibility testing are needed to validate these findings.

In summary, ST5 is the dominant sequence type of *C. neoformans* causing AIDS patients with CM in southern China. While clinical characteristics were similar across sequence type, infections with non-ST5 strains were associated with poorer outcomes, suggesting that sequence typing may have potential prognostic value in these patients.

## Figures and Tables

**Table 1:** Primer sequences of seven housekeeping genes used for MLST analysis.

	Primer Name	Primer Sequence
CAP59	CAP59F 5′	CTCTACGTCGAGCAAGTCAAG 3′
	CAP59R 5′	TCCGCTGCACAAGTGATACCC 3′
GPD1	GPD1F 5′	CCACCGAACCCTTCTAGGATA 3′
	GPD1R 5′	CTTCTTGGCACCTCCCTTGAG 3′
IGS1	IGSF 5′	ATCCTTTGCAGACGACTTGA 3′
	IGSR 5′	GTGATCAGTGCATTGCATGA 3′
LAC1	LAC1F 5′	AACATGTTCCCTGGGCCTGTG 3′
	LAC1R 5′	ATGAGAATTGAATCGCCTTGT 3′
PLB1	PLB1F 5′	CTTCAGGCGGAGAGAGGTTT 3′
	PLB1R 5′	GATTTGGCGTTGGTTTCAGT 3′
SOD1	SOD1F 5′	AAGCCTCTCATCCATATCTT 3′
	SOD1R 5′	TTCAACCACGAATATGTA 3′
URA5	URA5F 5′	ATGTCCTCCCAAGCCCTCGAC 3′
	URA5R 5′	TTAAGACCTCTGAACACCGTACTC 3′

**Table 2:** Allelic Profiles of MLST Loci in *Cryptococcus neoformans* Sequence Types.

ST	CAP59	GPD1	LAC1	PLB1	SOD1	URA5	IGS1
4	1	1	4	2	1	5	1
5	1	3	5	2	1	1	1
31	1	1	3	2	1	1	10
93	1	23	3	4	1	1	10
395	1	25	5	2	1	1	1
685	25	1	3	2	1	1	60
43	2	9	8	11	11	4	14

**Table 3:** Clinical Characteristics of *Cryptococcus neoformans* ST5 and non-ST5 types in AIDS Patients with CM.

Manifestations	Total (n = 100)	ST5 (n = 83)	Non-ST5 (n = 17)	Statistical Value	*p*
Gender, male (%)	80 (80.0%)	66 (79.5%)	14 (82.4%)	0	1.000
Age, year, median (IQR)	40 (30~47)	39.6 ± 11.4	37.8 ± 10.0	−0.675	0.542
Clinical symptoms					
Fever (%)	58 (58.0%)	46 (55.4%)	12 (70.6%)	1.332	0.248
Headache (%)	85 (85.0%)	71 (85.5%)	14 (82.4%)	0	1.000
Nausea/vomiting (%)	56 (56.0%)	47 (56.6%)	8 (47.1%)	1.356	0.470
Convulsion (%)	9 (9.0%)	5 (6.0%)	4 (23.5%)	3.358	0.067
Disturbance of consciousness (%)	23 (23.0%)	18 (21.7%)	5 (29.4%)	0.139	0.709
Cranial nerve damage (%)	21 (21.0%)	17 (20.5%)	4 (23.5%)	0	1.000
Hearing loss (%)	4 (4.0%)	3 (3.6%)	1 (5.9%)	-	0.531
Meningeal irritation (%)	60 (60.0%)	47 (56.6%)	13 (76.5%)	2.315	0.128
Time to diagnosis from symptom onset, (day), median (IQR)	18 (10~30)	18 (10~30)	16 (9~30)	−0.954	0.340
Cryptococcal encephalitis (%)	11 (11.0%)	7 (8.4%)	4 (23.5%)	1.923	0.165
Laboratory data					
CD4 count(cells/μL), median (IQR)	12 (5~34)	12 (5~34)	13 (5~35)	−0.106	0.916
CD4^+^/CD8^+^, median (IQR)	0.0 (0.02~0.07)	0.0 (0.02~0.08)	0.03 (0.02~0.07)	−0.731	0.465
CSF ICP (mmH_2_O)	165 (105~300)	165 (105~270)	243 (165~523)	−1.774	0.076
Brain CT/MRI				0.723	0.810
Normal (%)	56 (56.0%)	47 (74.6%)	9 (69.2%)		
Hydrocephalus (%)	2 (2.0%)	2 (3.2%)	0 (0.0%)		
Mass lesion (%)	18 (18.0%)	14 (22.2%)	4 (30.8%)		
Antifungal agent MIC					
5-FC S (%)	94 (98.9%)	80 (98.8%)	14 (100%)	-	1.000
I/R (%)	1 (1.1%)	1 (1.2%)	0 (0.0%)		
AMB S (%)	69 (71.9%)	56 (69.1%)	13 (86.7%)	1.155	0.283
I/R (%)	27 (28.1%)	25 (30.9%)	2 (13.3%)		
FCA S (%)	79 (82.3%)	68 (84.0%)	11 (73.3%)	0.386	0.534
I/R (%)	17 (17.7%)	13 (16.0%)	4 (26.7%)		
ITR S (%)	41 (59.4%)	34 (59.6%)	7 (58.3%)	1.193	0.275
I/R (%)	28 (40.6%)	23 (40.4%)	5 (41.7%)		
VRC S (%)	59 (86.8%)	50 (87.7%)	9 (81.8%)	0.002	0.966
I/R (%)	9 (13.2%)	7 (12.3%)	2 (18.2%)		

CSF, cerebrospinal fluid; ICP, intracranial pressure; CT, Computer tomography; MRI, Magnetic resonance imaging; S, Susceptible; I, Intermediate; R, Resistance; 5-FC, 5-flucytosine; AMB, Amphotericin B; FCA, Fluconazole; ITR, Itraconazole; VRC, Voriconazole.

## Data Availability

All relevant data are within the manuscript and its Supplementary Materials.

## Supplementary Materials

The following supporting information can be downloaded at: https://www.mdpi.com/article/10.3390/pathogens15060605/s1, Table S1: Survival by ST type at the 4-, 12-, 24-, and 48-week follow-up time points.
