# Supplementary material for: Sequence Types of Cryptococcus neoformans and Their Associations with Clinical Characteristics and Outcomes of AIDS Patients with Cryptococcal Meningitis in Southern China
Source: Pathogens. 2026 Jun 5;15(6):605. doi: 10.3390/pathogens15060605 (PMC13305016; doi:10.3390/pathogens15060605)
Supplement: Supplementary file 1 [file pathogens-15-00605-s001.zip › pathogens-4321687-supplementary.pdf]

Table S1. Survival by ST type at the 4-, 12-, 24-, and 48-week follow-up time points.

	**4-Week**			**12-Week**			**24-Week**			**48-Week**		
	**Survival**	**Non-survival**	***P***	**Survival**	**Non-survival**	***P***	**Survival**	**Non-survival**	***P***	**Survival**	**Non survival**	***P***
**STs**	84(84.0%)	16(16.0%)	0.263	67(67.0%)	33(33.0%)	**0.038**	63(63.0%)	37(37.0%)	0.089	61(61.0%)	39(39.0%)	0.123
**ST5**	72(85.7%)	11(68.8%)		60(89.6%)	23(69.7%)		56(88.9%)	27(73.0%)		54(88.5%)	29(74.4%)	
**ST4**	3(3.6%)	2(12.5%)		2(3.0%)	3(9.1%)		2(3.2%)	3(8.1%)		2(3.3%)	3(7.7%)	
**ST93**	2(2.4%)	2(12.5%)		0(0)	4(12.1%)		0(0)	4(10.8%)		0(0)	4(10.3%)	
**ST685**	3(3.6%)	0(0)		2(3.0%)	1(3.0%)		2(3.2%)	1(2.7%)		2(3.3%)	1(2.6%)	
**ST31**	2(2.4%)	1(6.3%)		2(3.0%)	1(3.0%)		2(3.2%)	1(2.7%)		2(3.3%)	1(2.6%)	
**ST43**	1(1.2%)	0(0)		1(1.5%)	0(0)		1(1.6%)	0(0)		1(1.6%)	0(0)	
**ST395**	1(1.2%)	0(0)		0(0)	1(3.0%)		0(0)	1(2.7%)		0(0)	1(2.6%)	

Survival by ST type, with *P* values indicating the probability that the distribution of ST types is significantly different between the survival and non-survival groups at each survival time. STs = sequence types.
